# Effects of Different Aerobic Exercises on Blood Lipid Levels in Middle-Aged and Elderly People: A Systematic Review and Bayesian Network Meta-Analysis Based on Randomized Controlled Trials

**DOI:** 10.3390/healthcare12131309

**Published:** 2024-06-30

**Authors:** Yuan Li, Qun Zhai, Ge Li, Weihang Peng

**Affiliations:** 1 Faculty of Health Sciences and Sports, Macao Polytechnic University, Macao SAR, China; p2313845@mpu.edu.mo; 2 School of Economics and Management, Chang’an University, Xi’an 710064, China; 2021123073@chd.edu.cn; 3 Faculty of Applied Sciences, Macao Polytechnic University, Macao SAR, China; p2311702@mpu.edu.mo

**Keywords:** aerobic exercise, blood lipids, middle-aged and elderly people, randomized controlled trial, Bayesian network meta-analysis

## Abstract

With increasing age, dyslipidemia becomes a common health problem in the middle-aged and elderly population, posing a significant risk of cardiovascular disease. Aerobic exercise, as a non-pharmacological intervention, is considered to be effective in improving blood lipid levels, but the extent to which different types of aerobic exercise affect blood lipids is not clear. This study aims to investigate the effects of 12 different aerobic exercises on total cholesterol, triglyceride, high-density lipoprotein cholesterol, and low-density lipoprotein cholesterol in middle-aged and elderly people aged 45 years and over through systematic review and Bayesian network Meta-analysis of randomized controlled trials. We systematically searched relevant databases and included eligible randomized controlled trials. Bayesian network meta-analysis was used to compare the effects of 12 types of aerobic exercise on lipid levels. A total of 487 randomized controlled trials involving middle-aged and elderly people over 45 years old were included. The results of the network meta-analysis showed that all types of aerobic exercise could reduce blood lipid levels compared with no intervention in middle-aged and elderly people. In terms of total cholesterol, triglyceride, and low-density lipoprotein cholesterol, swimming had the most significant effect. For HDL cholesterol, dance showed a better effect. Studies have shown that swimming and dancing have a positive effect on improving blood lipid levels in middle-aged and elderly people. It is recommended to choose the appropriate type of exercise according to personal preference and physical condition.

## 1. Introduction

In the fields of medicine and public health, dyslipidemia has been widely recognized as an important risk factor for cardiovascular disease in middle-aged and elderly people [1,2,3,4,5]. With the aging of the population, how to effectively control blood lipid levels has become an urgent public health problem that needs to be solved. Aerobic exercise, as a non-pharmacological intervention to improve blood lipids, has been widely considered because of its high safety and few side effects [6,7,8,9,10]. In modern society, with the acceleration of the pace of life and the change of lifestyle, the problem of dyslipidemia in middle-aged and elderly people has become increasingly prominent. The imbalance of blood lipid levels is not only a major risk factor for cardiovascular disease, but also a key factor leading to the increase of mortality and disability rates in middle-aged and elderly people [11,12,13]. Therefore, exploring effective blood lipid regulation methods is of great significance for improving the quality of life and prolonging the life span of middle-aged and elderly people.

Although drug therapy is the conventional means of adjusting lipid levels, long-term dependence on drugs may bring side effects and economic burden [14]. In addition, individual responses to drugs vary, making treatment effects difficult to predict. Therefore, finding a safe, effective and economical method for lipid management has become a current problem. Previous studies have demonstrated that aerobic exercise is effective in reducing total cholesterol (TC) and low-density lipoprotein cholesterol (LDL-C) while increasing high-density lipoprotein cholesterol (HDL-C). However, most of these studies have focused on a single type of aerobic exercise, and relatively few have comprehensively compared and evaluated different types of aerobic exercise [15,16,17,18,19,20,21,22,23].

Dyslipidemia not only increases the risk of cardiovascular disease in middle-aged and elderly people, but also causes a variety of chronic diseases such as diabetes and hypertension, which seriously affects the quality of life of patients. In addition, health problems caused by dyslipidemia can increase the burden on the healthcare system and cause huge socio-economic losses. Although the positive effects of aerobic exercise on lipids are well established, the exact type of aerobic exercise that is most effective and the extent to which different exercise modalities affect specific lipid measures remain challenging research. In addition, the physical conditions and exercise adaptability of middle-aged and elderly people are quite different, and how to provide them with personalized exercise advice is also a challenge.

Aerobic exercise, as a lifestyle intervention, is widely recognized to improve lipid levels and reduce the risk of cardiovascular disease. However, the results of studies on the effects of different aerobic exercises on blood lipid levels are inconsistent, and there is a lack of systematic evaluation and comparison. Traditional meta-analyses can improve statistical power but are limited when multiple treatments are involved. Network meta-analysis integrates direct and indirect evidence, providing a more comprehensive comparison of treatment effects. This improves the statistical efficiency of the study and allows us to obtain more precise effect estimates. Such precision is critical for clinical decision-making and policymaking [24]. Through systematic review of randomized controlled trials and Bayesian network Meta-analysis, this study comprehensively compared and evaluated the effects of 12 different aerobic exercises on total cholesterol, triglyceride, high-density lipoprotein cholesterol, and low-density lipoprotein cholesterol levels in middle-aged and elderly people over 45 years old. Our study not only fills the gaps in the existing literature, but also provides more scientific and personalized exercise guidance for middle-aged and elderly people. Through the comprehensive evaluation of different types of aerobic exercise, this study will provide more accurate guidance for the blood lipid management of middle-aged and elderly people, help to reduce the incidence of cardiovascular disease, improve the quality of life, and promote the overall improvement of social health.

## 2. Materials and Methods

### 2.1. Registration

The study complied with PRISMA guidelines [25]. In addition, this study followed the Cochrane Handbook for Systematic Reviews of Interventions, and the protocol has been registered in the International Registry of prospective Systematic Reviews PROSPERO database under registration number: CRD42024519466.

### 2.2. Data Sources and Search Strategies

In this study, databases from Web of Science, PubMed, EBSCO, and Scopus were searched from the beginning of database construction to 2024, and the search language was English. Literature screening and data extraction were completed by two researchers independently. According to the inclusion and exclusion criteria, each researcher read the title and abstract, screened the relevant studies, and read the full text of the included studies. If two investigators disagreed on a study, the parties discussed and exchanged views or inclusion was decided by a third researcher. The final extracted content of the study included the name of the researcher, publication time, mean value, standard deviation, sample size, etc. It was identified after repeated pre-examination and supplemented by manual search, and references of included articles were traced back if necessary.

The search strategy was created using a combination of medical subject headings (Mesh) terms, keywords, and phrases. The search terms were aerobic exercise, randomized controlled trial, middle-aged, elderly, exercise, and all results from these databases were exported to EndNote20.4 software. PubMed was used as an example. The search formula is shown in Table 1.

### 2.3. Inclusion Criteria and Exclusion Criteria

#### 2.3.1. Inclusion Criteria

The inclusion criteria strategy was defined according to subjects, interventions, comparisons, outcomes and study design (PICOS). (1) Subjects were men and women, with an average age of 45 years and above, and clinically diagnosed with poor health status, dyslipidemia, or chronic disease to ensure the applicability and pertinences of the study results. (2) The included studies were randomized controlled trials to reduce the influence of bias and confounding factors. The control group received non-exercise intervention (NEI). (3) At least one of four lipid markers (total cholesterol, triglyceride, low-density lipoprotein cholesterol, or high-density lipoprotein cholesterol) was included; to be included, studies had to include aerobic exercise as the primary intervention and the exercise had to meet the definition of aerobic exercise as continuous, rhythmic, and moderate intensity.

#### 2.3.2. Exclusion Criteria

Exclusion criteria included the following: (1) studies that were not randomized controlled trials, such as observational studies, cohort studies, cross-sectional studies, and case-control studies, were excluded to avoid methodological bias; (2) studies with a single aerobic exercise intervention or an undefined type of aerobic exercise intervention, which were excluded to ensure the consistency and comparability of the interventions; (3) reviews of the literature; (4) studies that could not provide complete original data or statistical analysis results, to ensure the accuracy and credibility of the results; (5) combined intervention trials; (6) studies with non-middle-aged and elderly subjects; (7) studies with a control group that did not meet our requirements, such as drug control; (8) studies with incorrect data or missing literature.

### 2.4. Data Collection

The following data were collected: (1) basic information, including the first author’s name and publication year; (2) the demographic characteristics of the subjects, such as gender and age; (3) information on study design, such as sample size, interventions, measurement parameters, follow-up time, and information related to risk of bias assessment.

### 2.5. Quality Evaluation

The Cochrane recommended risk assessment tool RevMan5.4 was used to assess the quality of the included studies, including random sequence generation, assignment concealment, blinding, incomplete outcome data, selective reporting, and other biases. The literature quality was divided into three levels from high to low: low risk, medium risk, and high risk. If the above quality evaluation criteria are fully met, the study has a low-risk level and the possibility of bias is small. If the above quality evaluation criteria are partially met, the risk is medium and the possibility of bias is moderate. If the above quality evaluation criteria are not met at all, the study is high risk and the possibility of bias is high.

### 2.6. Statistical Analysis

RevMan5.4, Stata 15, and R3.6.2 software was used for network meta-analysis. The RevMan5.4. Software recommended by Cochrane was used to evaluate the quality of the literature. Stata15 software was used to draw the network structure diagram and funnel plot. The initial value was set through four Markov chains, with an initial value of 2.5, and 20,000 iterations for annealing plus another 50,000 iterations to achieve model convergence, with a step size of 1.

The deviance information criterion (DIC) was used to evaluate the overall inconsistency by comparing the fit of the consistency model and the inconsistency model [26]. When the potential scale reduction factors (PSRF) were close to 1, the convergence degree of the model was satisfactory, and the Rank chart was drawn to predict the efficacy of each intervention. When there was a closed loop, the node splitting method was used to detect the inconsistency between direct evidence and indirect evidence, and the *p* value was calculated. If the *p* value was >0.05, there was no inconsistency between each node. The odds ratio (OR) was used as the effect analysis statistic for binary variables, and the mean difference (MD) was used as the effect analysis statistic for continuous variables. A 95% confidence interval (CI) was provided for each effect size [27]. The overall ranking of treatments was estimated by calculating the area under the cumulative ranking probability map (SUCRA) value of each method, and the merits of interventions were ranked according to the SUCRA value.

## 3. Results

### 3.1. Study Selection

**Figure 1:**
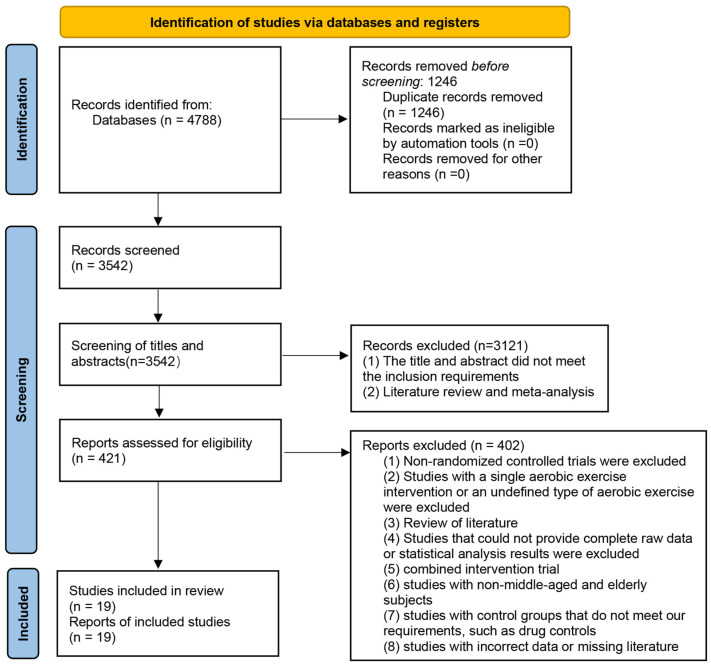
PRISMA flow diagram of study selection.

According to the flow chart of PRISMA, a total of 4788 relevant studies were retrieved. After duplication removal, title and abstract screening, and full text screening, 19 eligible RCTS were finally included. The flow chart of the study screening is shown in Figure 1.

### 3.2. Study Characteristics and Literature Quality Evaluation

#### 3.2.1. Characteristics

Nineteen studies involving 487 subjects were included [28,29,30,31,32,33,34,35,36,37,38,39,40,41,42,43,44,45,46]. All subjects were middle-aged and elderly, with a mean age of over 45 years. Twelve different aerobic exercise interventions were included in the study. The control group was the non-exercise intervention group (NEI). The intervention content was aerobic exercise. All studies were randomized controlled trials, and the 19 included studies were continuous variable studies. There were 11 studies with an experimental period of more than 40 h and six studies with one of less than 40 h. Two studies did not report detailed timing of intervention. Among the included studies, there were 14 studies with the outcome indicators of total cholesterol, triglyceride, high-density lipoprotein cholesterol, and low-density lipoprotein cholesterol. One study did not report the outcome indicators of total cholesterol, and four studies did not report the outcome indicators of low-density lipoprotein cholesterol. Two studies did not report outcome measures of high-density lipoprotein cholesterol. None of the 19 studies reported any adverse effects. The main characteristics of each included study are shown in Table 2.

#### 3.2.2. Quality Assessment

**Figure 2:**
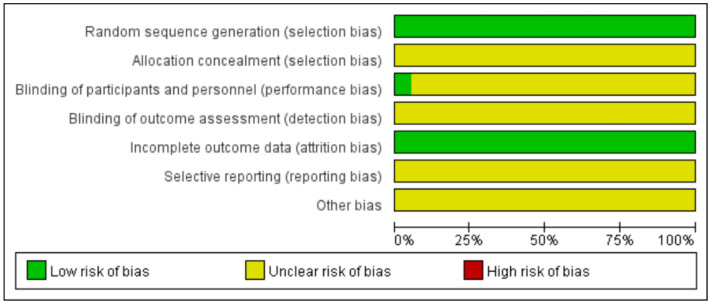
Risk of bias graph of included studies.

**Figure 3:**
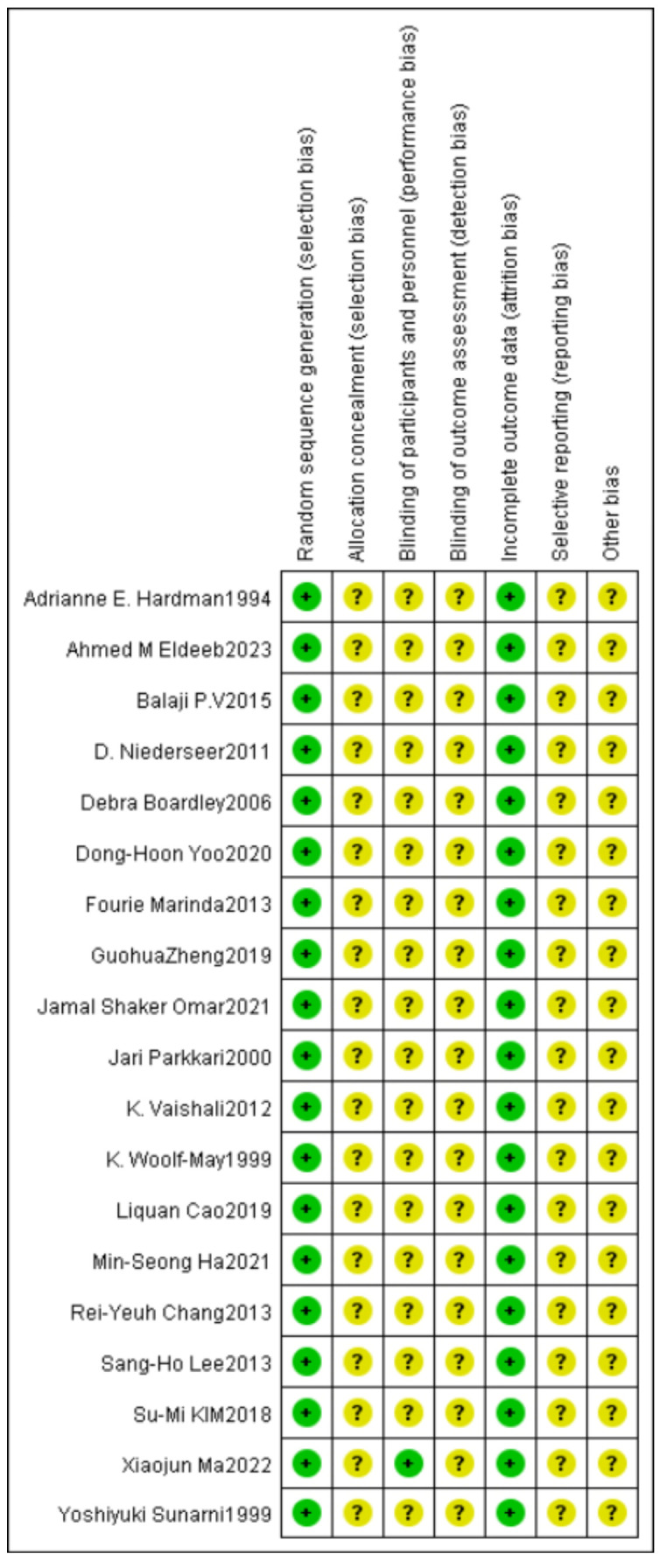
Summary of the risk of bias of included studies.

In this study, the quality of the included literature was evaluated using the evaluation principles of the Cochrane Center for Evidence-based Medicine for randomized controlled trials, and the results showed that 19 studies were generated using random sequences and rated as low risk. Regarding allocation concealment and blinding, one study had a single-blind design, and the remaining studies were not mentioned. The risk of incomplete outcome data was low. In terms of selective reporting, protocols were not available for all studies, so none of the risks of bias were known. Other sources of bias were unclear, and the quality of the included literature was generally high, but there was some ambiguity in terms of implementor blinding, outcome assessor blinding, other biases, etc. Detailed results are shown in Figure 2 and Figure 3.

### 3.3. Results of Network Meta-Analysis

#### 3.3.1. Evidence Network Relationship

**Figure 4:**
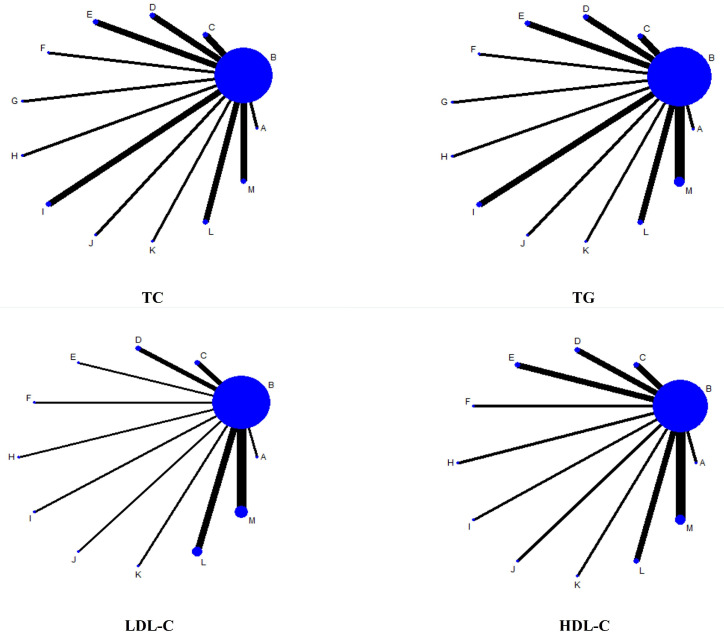
Evidence network diagram. Note: TC = total cholesterol; TG = triglycerides; HDL-C = high-density lipoprotein cholesterol; LDL-C = low-density lipoprotein cholesterol.

The connection between the line points indicates the presence of direct comparative evidence between the networks. Letters outside the line indicate different interventions. In the network plots of the effects of 12 interventions, including the control group, on blood lipids in middle-aged and elderly people, the area of the circles represents the size of the sample size of the corresponding intervention study. The thickness of the connecting line indicates the strength of the comparison between the different interventions. The detailed results are shown in Figure 4.

#### 3.3.2. Consistency Test and Convergence Diagnosis

The results of the DIC values are as follows; where the absolute value of DIC differs by less than 5, and the degree of fit of the two models is considered to be consistent. The PSRF of the operation is close to 1, and its convergence effect is very good. The DIC results for each model are shown in Table 3.

#### 3.3.3. Effect on Total Cholesterol

**Figure 5:**
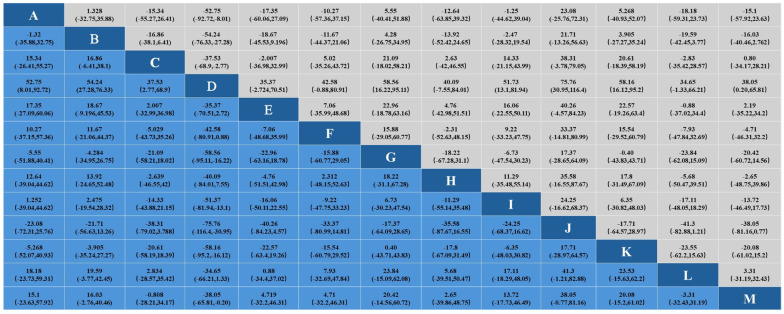
Results of network Meta-analysis.

**Figure 6:**
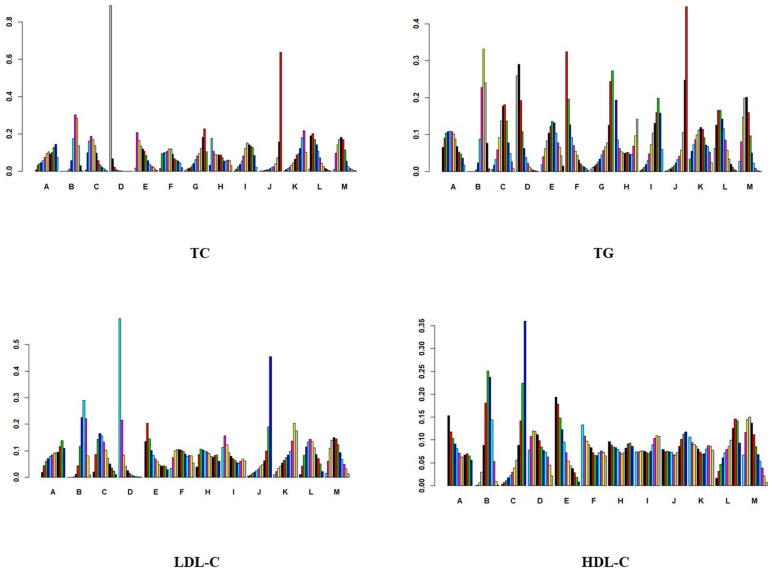
Probability ranking diagram.

**Figure 7:**
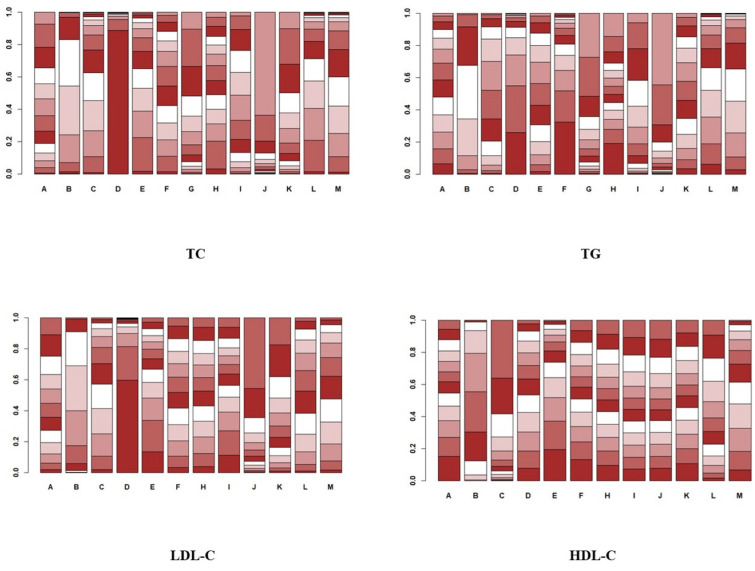
Cumulative probability ranking plot.

In the study of the effect of aerobic exercise on blood lipids in middle-aged and elderly people, a total of 18 studies reported total cholesterol levels in 937 subjects. Intervention D (swimming) showed statistical differences in total cholesterol reduction compared with A (cycling), B (non-exercise intervention), C (yoga), G (Pilates), I (brisk walking), J (skiing), K (golf), and M (Baduanjin), indicating that its effect on lowering total cholesterol levels may be the most significant. No significant difference was found in other interventions. The specific results are shown in Figure 5. In the analysis of the effect of the aerobic exercise intervention on total cholesterol, we calculated the rank probability of each intervention to assess its likelihood of being the best treatment. The SUCRA value was calculated to comprehensively evaluate the relative effectiveness of each intervention. A higher SUCRA value indicates that the intervention performs better in all comparisons. Figure 6 and Figure 7 show a probability ranking rank graph and cumulative ranking graph.

**Figure 8:**
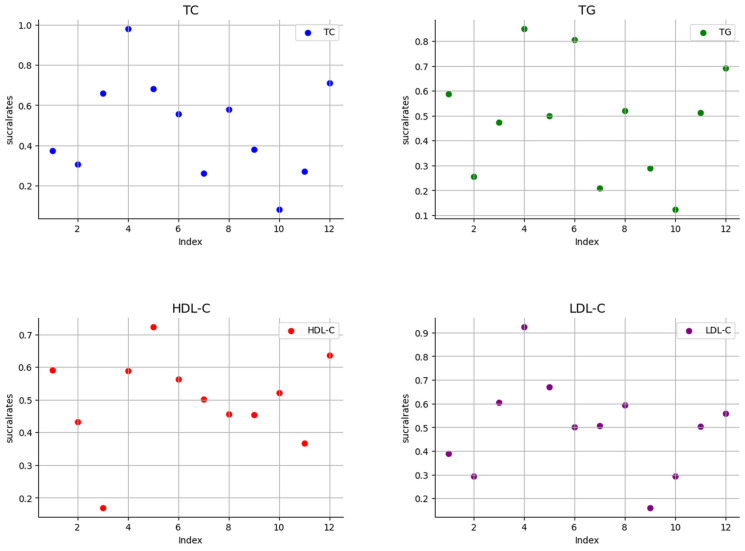
Scatter plot of SUCRA values.

The ranking results show that swimming is most effective in reducing total cholesterol; the specific results are shown in Figure 6 and Figure 7. The SUCRA results, as illustrated in Figure 8, show that swimming is the most effective intervention for reducing total cholesterol levels. The results of SUCRA ranking show the following: swimming (0.98) > walking (0.70) > yoga (0.6600) > Baduanjin (0.6608) > dance (0.68) > running (0.57) tai chi (0.55) > brisk walking (0.379) > cycling (0.371) > non-exercise intervention (0.30) > golf (0.269) > Pilates (0.260) > skiing (0.08).

#### 3.3.4. Effects on Triglycerides

**Figure 9:**
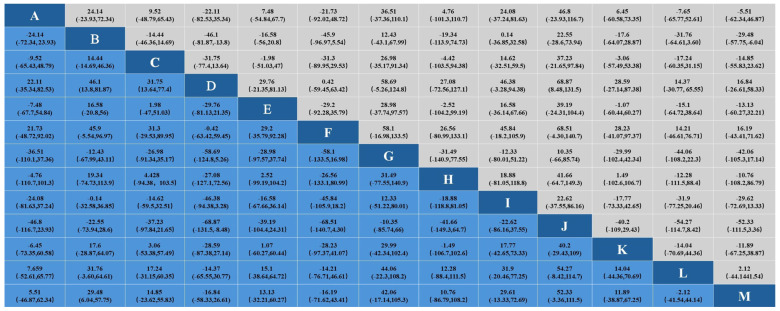
Results of network Meta-analysis.

In the study of the effect of aerobic exercise on blood lipids in middle-aged and elderly people, a total of 19 studies reported triglyceride levels in 997 subjects. The results of the Bayesian network meta-analysis showed that intervention D (swimming) showed statistically significant differences in triglyceride reduction compared with intervention B (non-exercise intervention) and J (skiing). Indicates that its effect in reducing triglyceride may be the most significant. No significant difference was found in other interventions. The specific results are shown in Figure 9.

In the analysis of the effect of the aerobic exercise intervention on triglycerides, we calculated the ranking probability of each intervention to assess its likelihood of being the best treatment. The SUCRA value was calculated to comprehensively evaluate the relative effectiveness of each intervention. A higher SUCRA value indicates that the intervention performs better in all comparisons. The ranking results of probability ranking rank chart and cumulative ranking chart show that swimming is the first in reducing triglyceride. The specific results are shown in Figure 6 and Figure 7. The SUCRA results, as illustrated in Figure 8, show that swimming is the most effective intervention for reducing triglyceride levels. The ranking results of SUCRA show the following: swimming (0.85) > tai chi (0.80) > Baduanjin (0.68) > walking (0.69) > cycling (0.58) > golf (0.51) > running (0.52) > yoga (0.47) > dance (0.49) > brisk walking (0.28) > non-exercise intervention (0.25) > Pilates (0.20) > skiing (0.12).

#### 3.3.5. Effect on Low-Density Lipoprotein Cholesterol

**Figure 10:**
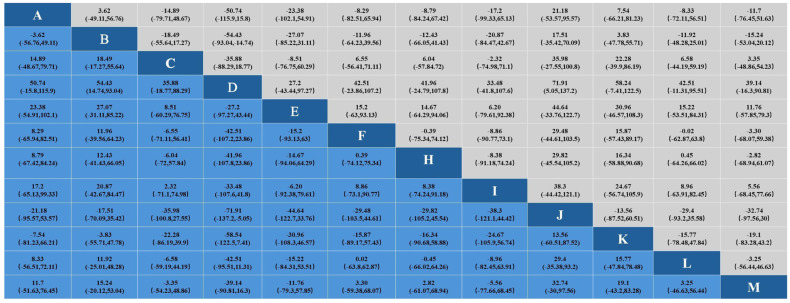
Results of network Meta-analysis.

In the study of the effects of aerobic exercise on blood lipids in middle-aged and elderly people, a total of 15 studies reported low-density lipoprotein cholesterol levels in 854 subjects. The results of the Bayesian network Meta-analysis showed that intervention D (swimming) compared with B (non-exercise intervention) and J (skiing), and intervention D (swimming) compared with intervention B (non-exercise intervention) and J (skiing), showed significant differences. A statistical difference was shown in lowering LDL cholesterol, suggesting that its effect in lowering LDL total cholesterol levels may be most pronounced. No significant difference was found in other interventions. The specific results are shown in Figure 10.

In the analysis of the effect of the aerobic exercise intervention on low-density lipoprotein cholesterol, we calculated the rank probability of each intervention to assess its likelihood of being the best treatment. The SUCRA value was calculated to comprehensively evaluate the relative effectiveness of each intervention. A higher SUCRA value indicates that the intervention performs better in all comparisons. The ranking results of probability ranking rank chart and rank probability ranking chart show that swimming is the most effective at reducing low-density lipoprotein cholesterol, and the specific results are shown in Figure 6 and Figure 7. The SUCRA results, as illustrated in Figure 8, indicate that swimming is the best intervention for lowering LDL cholesterol. The ranking results of SUCRA show the following: swimming (0.92) > dance (0.67) > yoga (0.60) > brisk walking (0.59) > Baduanjin (0.55) > running (0.505) walking (0.504) > tai chi (0.50) > cycling (0.39) > non-exercise intervention (0.294) > golf (0.292) > skiing (0.16).

#### 3.3.6. Effect on High-Density Lipoprotein Cholesterol

In the study of the effect of aerobic exercise on blood lipids in middle-aged and elderly people, a total of 17 studies reported the total cholesterol level of 915 subjects. The Bayesian network Meta-analysis was conducted, and the results showed that there was no statistical difference between different intervention measures. This finding may be due to the similar effects of the different interventions on HDL cholesterol levels. The specific results are shown in Figure 10.

**Figure 11:**
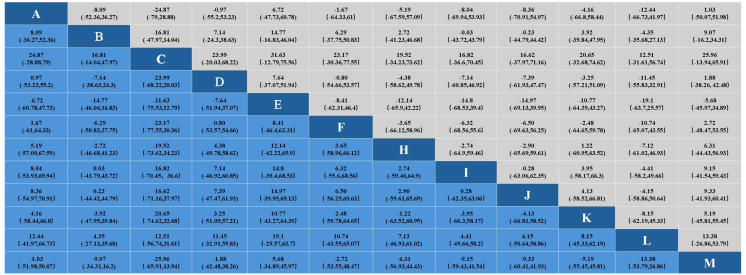
Results of network Meta-analysis.

In the analysis of the effect of the aerobic exercise intervention on high-density lipoprotein cholesterol, we calculated the rank probability of each intervention to assess its likelihood of being the best treatment. The SUCRA value was calculated to comprehensively evaluate the relative effectiveness of each intervention. A higher SUCRA value indicates that the intervention performs better in all comparisons. The results of the probability ranking chart and the cumulative probability ranking chart showed that dance was the most effective at increasing high-density lipoprotein cholesterol. The specific results are shown in Figure 6 and Figure 7. The SUCRA results, as illustrated in Figure 8, show that dance is the most effective intervention for increasing HDL cholesterol levels. The results of SUCRA ranking show the following: dancing (0.72) > Baduanjin (0.63) > cycling (0.589) > swimming (0.587) > tai chi (0.56) > golf (0.52) running (0.50) > brisk walking (0.456) > skiing (0.453) > non-exercise intervention (0.43) > walking (0.36) > yoga (0.16) (Figure 11).

#### 3.3.7. Assessment of Publication Bias

**Figure 12:**
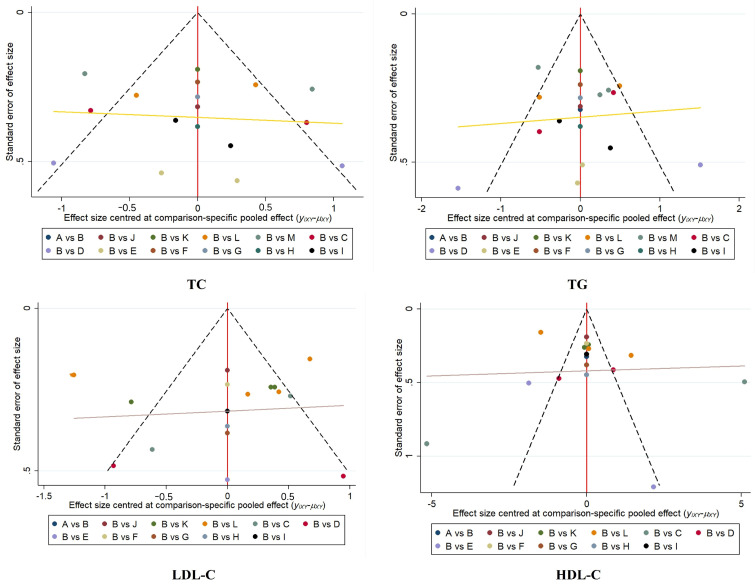
Plot of funnel.

The funnel plot showed that the study points were symmetrically distributed at the top of the funnel plot and were relatively evenly distributed; however, there were a few points that deviated from the expected distribution, suggesting that we need to further investigate the potential bias of these studies. Despite individual study deviations, no significant publication bias was observed overall. The specific results are shown in Figure 12.

## 4. Discussion

In this study, we conducted a systematic review and Bayesian network meta-analysis of randomized controlled trials to investigate the effects of 12 different aerobic exercises on lipid levels in middle-aged and elderly people aged 45 years and older. The results showed that all types of aerobic exercise could significantly lower blood lipid levels compared with no intervention. Among them, swimming, as a systemic aerobic exercise, was the most effective in reducing total cholesterol (TC), low-density lipoprotein cholesterol (LDL-C), and triglyceride (TG) in middle-aged and older adults. This may be due to the fact that the buoyancy of water during swimming reduces joint pressure, allowing middle-aged and elderly people to exercise for a longer period of time, thereby promoting lipid metabolism more effectively. F. H. Abadi et al. (2020), N. Nualnim et al. (2012) [47,48].

In addition, dance was shown to be more effective for raising HDL cholesterol. Dance, as a rhythmic and social exercise, not only improves HDL cholesterol, but also improves mental health and quality of life. The variety and fun of dance makes middle-aged and elderly people more willing to continue to participate and, as such, to obtain improvements in blood lipid levels in the long term. Related studies also show that for middle-aged and elderly people, dance has a certain social role. Lynn O T’oole et al. (2015) found a significant difference in activity participation frequency (*p* = 0.036) after the dance program, and the data indicated that participants liked the program and perceived enhanced physical ability, improved emotional and mental health, and increased activity as a result of participation in the program [49]. Therefore, this positive emotion can enable middle-aged and elderly people to continue to participate in aerobic exercise, so as to obtain improvements in blood lipid levels in the long term. These findings have important implications for health management in middle-aged and elderly populations and fill gaps in the existing literature.

At present, relevant studies mostly focus on the effects of a single exercise on blood lipid levels, and there is a lack of comparison of the effects of different exercise on blood lipid levels. Most of the studies on the effects of different exercise on blood lipid levels in middle-aged and elderly people are based on frequency-based network meta-analysis, not based on Bayesian theorem, which may bias the research results. Firstly, our study conducted a network meta-analysis based on Bayesian theorem, which was the first time that Bayesian network meta-analysis was applied to the research on the effects of different aerobic exercises on blood lipids. It provided a new analysis framework for future related research, further improved the research results from statistics, and ensured the stability of the results. Y. Buzdagli et al. (2022) and Hezhang Yun et al. (2023) found that aerobic exercise had a significant effect on blood lipid levels compared with other types of exercise, but the study did not say which aerobic exercise had a more significant effect on blood lipid levels. Moreover, the effect of aerobic exercise on blood lipid levels in middle-aged and elderly people was unclear [50,51]. Yanan Gao et al. (2021) found that traditional Chinese exercise had an effect on blood lipid levels in middle-aged and elderly people, but this study did not include most mainstream aerobic exercises popular in current society and was not comprehensive [52]. Mahdi Ghafari et al. (2020) found the effects of different exercises on blood lipid levels in the elderly through meta-analysis. This study included people under the average age of 45 years old, and the study was not rigorous [53]. On the basis of these studies, our study covers a variety of exercise types and is based on randomized controlled trials, which provides empirical data support for the study and enhances the credibility of the findings. In addition to the traditional Chinese aerobic exercise, we also added the current mainstream aerobic exercise to conduct Bayesian network meta-analysis, which enhanced the credibility of the study results. It makes up for the deficiencies of the above studies.

Our study provides precise guidance for middle-aged and elderly people, which will help to provide more precise guidance for the management of blood lipids in middle-aged and elderly people and promote the overall improvement of social health. The promotion of effective aerobic exercise may reduce the incidence of cardiovascular disease and improve the quality of life in middle-aged and elderly people. At the same time, it can provide a scientific basis for the formulation of public health policies for middle-aged and elderly people, especially in the promotion of non-pharmaceutical interventions.

However, there are some limitations to this study. First, the number of included studies was limited and the sample size was relatively small, which may have affected the generalizability and reliability of the results. Second, because of the lack of long-term follow-up data, we could not determine the long-term effects of aerobic exercise on lipid levels. In addition, this study failed to take into account factors such as the lifestyles, dietary habits, and genetic backgrounds of different individuals, all of which may affect the effect of aerobic exercise on lipid levels. Future studies with larger sample sizes and long-term follow-up should assess the long-term effects of aerobic exercise on lipid levels and account for the contribution of individual differences. In addition, studies should explore the effects of aerobic exercise with different intensities and frequencies on lipid levels, in order to provide more precise and personalized exercise recommendations for middle-aged and older adults.

In conclusion, the results of this study highlight the important role of aerobic exercise in lipid management in middle-aged and elderly people, and especially the potential of swimming and dancing to improve lipid levels. Aerobic exercise provides a safe, effective, and economical method for lipid management in middle-aged and elderly people, which helps to reduce the risk of cardiovascular disease and improve the quality of life.

## 5. Conclusions

Based on a systematic review of randomized controlled trials and Bayesian network meta-analysis, this study investigated the effects of 12 different types of aerobic exercise on total cholesterol, triglycerides, high-density lipoprotein (HDL) cholesterol, and low-density lipoprotein (LDL) cholesterol in middle-aged and elderly people aged 45 years and above. The results showed that all types of aerobic exercise could reduce lipid levels in middle-aged and elderly people, with swimming and dancing having the most significant effects. Swimming, in particular, showed statistically significant differences in lowering total cholesterol, triglycerides, and LDL cholesterol, while dancing performed better in raising HDL cholesterol. These findings provide more scientific and more personalized exercise guidance for middle-aged and elderly people, which can help to reduce the incidence of cardiovascular disease and improve the quality of life. Future studies should further explore the optimal type, intensity, and frequency of aerobic exercise to optimize lipid management strategies. Other factors, such as study quality and funding sources, should also be considered to improve the accuracy and reliability of meta-analyses. In conclusion, aerobic exercise, as an effective non-pharmacological intervention, has a positive effect on improving lipid levels in middle-aged and elderly people. It is recommended to choose the appropriate type of exercise according to personal preference and physical condition.

## Figures and Tables

**Table 1:** Search strategies in PubMed.

No.	Search Content
#1	“Middle Aged” [MeSH Terms]
#2	“Exercise” [MeSH Terms]
#3	“Aged” [MeSH Terms]
#4	“Randomized Controlled Trial” [Publication Type]
#5	“aerobic exercise” [Title/Abstract]
#6	“Middle Aged” [MeSH Terms] AND “Exercise” [MeSH Terms] AND “Aged” [MeSH Terms] AND “Randomized Controlled Trial” [Publication Type] AND “aerobic exercise” [Title/Abstract]

**Table 2:** A list of basic characteristics of the studies included in the meta-analysis.

Study ID and Time of Publication	Subjects	Sample Size						Age (Years)		Interventions		Duration of Intervention (Hours)	Outcome Measures *
		Experimental Group			Control Group			Experimental Group	Control Group	Experimental Group	Control Group		
		male	female	Total number	male	female	Total number						
Yoshiyuki Sunami (1999) [42]	Healthy	10	10	20	10	10	20	67 ± 4	68 ± 4	Bicycle (A)	NEI	60	1234
K. Vaishali (2012) [43]	Chronic diseases	14	3	27	22	8	30	65.8 ± 3.2	64.4 ± 3.8	Yoga (C)	NEI	72	1234
Balaji P.V (2015) [28]	Chronic diseases	None	None	15	None	None	15	45–60	45–60	Yoga (C)	NEI	60	1234
Min-Seong Ha (2018) [33]	Healthy	None	11	11	None	8	8	74.09 ± 4.21	76.00 ± 5.52	Swimming (D)	NEI	30	1234
Jamal Shaker Omar (2021) [40]	Chronic diseases	10	10	20	10	10	20	52.4 ± 5.5		Swimming (D)	NEI	96	1234
Dong-Hoon Yoo (2020) [45]	Healthy	8	None	8	8	None	8	70.88 ± 3.6	70.00 ± 2.39	Dance (E)	NEI	36	1234
Su-Mi KIM (2018) [35]	Healthy	None	7	7	None	6	6	78.0 ± 1.5	77.7 ± 1.6	Dance (E)	NEI	36	134
Rei-Yeuh Chang (2013) [31]	Chronic diseases	21	16	37	20	17	37	57.6 ±8.7	59.0 ±10.7	Tai Chi (F)	NEI	78	1234
Fourie Marinda (2013) [38]	Healthy	None	25	25	None	25	25	66.12 ± 4.77	65.32 ± 5.01	Pilates (G)	NEI	None	14
Liquan Cao (2019) [30]	Obesity	None	13	13	None	15	15	63.8 ± 5.9	64.0 ± 4.6	Running (H)	NEI	36	1234
K. Woolf-May (1999) [44]	Healthy	13	6	19	8	5	13	50.1 ± 6.3	54.7 ± 7.0	Brisk walking (I)	NEI	84	124
Adrianne E. Hardman (1994) [34]	Healthy	None	10	10	None	10	10	47.3 ± 2	41.6 ± 1.2	Brisk walking (I)	NEI	24	134
D. Niederseer (2011) [39]	Healthy	12	10	22	10	10	20	66.6 ± 2.1	67.3 ± 4.4	skiing (J)	NEI	112	1234
Jari Parkkari (2000) [41]	Chronic diseases	55	None	55	55	None	55	55 ± 4	55 ± 4	golf (K)	NEI	200	1234
Sang-Ho Lee (2013) [36]	Obesity	None	38	38	None	23	23	46.8 ± 3.1	46.7 ± 2.8	Walking (L)	NEI	None	1234
Debra Boardley (2006) [29]	Healthy	11	22	33	9	26	35	73.2 ± 6.6	75.9 ± 7.7	Walking (L)	NEI	40	1234
Ahmed M Eldeeb (2023) [32]	Metabolic syndrome	16	14	30	17	13	30	63.07 ± 3.17	62.8 ± 3.25	Baduanjin (M)	NEI	56	34
Guohua Zheng (2019) [46]	Healthy	30	55	85	31	54	85	60.53 ± 6.29	59.75 ± 6.34	Baduanjin (M)	NEI	60	1234
Xiaojun Ma (2022) [37]	Chronic diseases	16	18	34	14	18	32	59.18 ± 3.93	59.09 ± 5.25	Baduanjin (M)	NEI	182	1234

Note: NEI indicates no exercise intervention group; * Outcome measures: 1 = Total Cholesterol (TC), 2 = Low-Density Lipoprotein Cholesterol (LDL-C), 3 = High-Density Lipoprotein Cholesterol (HDL-C), and 4 = Triglycerides (TG).

**Table 3:** DIC results for each model.

Outcome Measures	Model of Consistency	Model of Inconsistency
TC	69.88	69.76
TG	73.37	73.39
LDL-C	59.33	59.43
HDL-C	67.72	67.80

Note: TC is total cholesterol; TG is triglyceride; LDL-C is low density lipoprotein cholesterol; HDL-C is high density lipoprotein cholesterol.

## Data Availability

The data that support the findings of the study are available from the first author, upon reasonable request.

## Abbreviations

A	Cycling
B	Non-exercise intervention (NEI)
C	Yoga
D	Swimming
E	Dance
F	Tai chi
G	Pilates
H	Running
I	Brisk walking
J	Skiing
K	Golf
L	Walking
M	Baduanjin
NEI	Non-exercise intervention;
TC	Total cholesterol;
TG	Triglycerides;
HDL-C	High-density lipoprotein cholesterol;
LDL-C	Low-density lipoprotein cholesterol;
RCT	Randomized controlled trials;
MD	Mean differences;
SUCRA	Surface under the cumulative ranking.
